# A fetus in the world: Physiology, epidemiology, and the making of fetal origins of adult disease

**DOI:** 10.1007/s40656-023-00598-z

**Published:** 2023-12-13

**Authors:** Tatjana Buklijas, Salim Al-Gailani

**Affiliations:** 1 Koi Tū: Centre for Informed Futures & Global Studies, The University of Auckland, Auckland, New Zealand; 2 Department of History and Philosophy of Science, University of Cambridge, Cambridge, UK

**Keywords:** DOHaD, Fetus, Epidemiology, Physiology, Pregnancy

## Abstract

Since the late 1980s, the *fetal origins of adult disease*, from 2003 *developmental origins of health and disease* (DOHaD), has stimulated significant interest in and an efflorescence of research on the long-term effects of the intrauterine environment. From the start, this field has been interdisciplinary, using experimental animal, clinical and epidemiological tools. As the influence of DOHaD on public health and policy expanded, it has drawn criticism for reducing the complex social and physical world of early life to women’s reproductive bodies as drivers of intergenerational ills. This paper explains this narrowing of focus in terms of a formative and consequential exchange between David Barker, the British epidemiologist whose work is credited with establishing the field, and the discipline of fetal physiology. We suggest that fetal physiologists were a crucial constituency of support for Barker’s hypothesis about early life origins of disease. Their collaborations with Barker helped secure and sustain the theory amid considerable controversy. The trajectory of DOHaD and its focus on the maternal body can be understood, we argue, as a consequence of this alliance, which brought together two distinct conceptualizations of the intrauterine environment, one from epidemiology and the other from fetal physiology. Along the way, we trace the histories of these conceptualizations, both of which were products of mid-to-late twentieth century British science, and show how Barker’s early emphasis on social and economic conditions was superseded by a narrower focus on physiological mechanisms acting upon the autonomous fetus.

In 1989 a small group of scientists from the United Kingdom, Italy, West Germany, Canada, and New Zealand met in the Ligurian countryside at a symposium titled *Fetal autonomy and adaptation*. Some were practicing clinicians; many experimental scientists; and all were affiliated with the field of fetal physiology. The meeting was not a regular get-together to discuss research progress, institutional and professional news. Rather, its key objective was to reflect on the state of the field and its future directions. The functions of the animal fetal body have been studied from the early days of experimental physiology, at German universities of the late nineteenth century (Cohnstein & Zuntz, 1884) but more systematically from the 1930s. In this period the field of fetal physiology developed around the laboratory of the Cambridge physiologist Joseph Barcroft (1947). From the 1960s onwards, they combined experimental work with new visual and other non-invasive methods of human fetal observation. But by the late 1980s the field leader for four decades, the Oxford physiologist Geoffrey Dawes (1918–1996), believed it needed fresh questions and approaches (Dawes et al., 1990). To do so, he invited David Barker from the MRC Environmental Epidemiology Unit at the University of Southampton. Barker had used the methods of historical demography and (environmental) epidemiology to argue that early life conditions set the developing organism on a path leading to adult health or chronic disease (Barker et al., 1986, 1987, 1989a, 1989b, 1990). This argument came to be known as “Barker’s hypothesis”.

When Barker and the physiologists first met at the Villa Marigola conference, their encounter was not immediately harmonious. Yet from 1990 onwards, they came together in *fetal origins of adult disease*, later *developmental origins of health and disease* (DOHaD), an interdisciplinary field using experimental animal, clinical, epidemiological, and—after 2005—molecular epigenetic tools to test and expand Barker’s hypothesis (Gluckman et al., 2016). Ever since, DOHaD has drawn criticism from a range of fields, including epidemiology, for unsatisfactory causal explanation and for the apparent distance from clinical relevance (Robinson, 1992; Penkler, 2022; Richardson, 2021). Nevertheless, the field has grown meteorically in significance and reach, as an important explanatory framework not only for scientists and clinicians but also in health policy first at the national, and then increasingly the global stage (Pentecost & Ross, 2019).

As the influence of DOHaD has grown, social scientists have critically assessed the field’s perceived reduction of the complex social and physical world of early life to the maternal body. In the process, they argued, it drew on historical, gendered concepts of maternal responsibility for the health of future generations, while simultaneously disregarding the effects of class, race and gender inequities, or other local contingencies that shaped people’s lives (Pentecost & Meloni, 2020; Richardson et al., 2014; Valdez, 2021; Warin et al., 2012). These critiques of DOHaD are now well known. Yet we have no clear account of how Barker, an environmental epidemiologist trained in social medicine, a discipline whose entire purpose was to understand the impact of social and economic conditions upon health, came to embrace such an individualistic, fragmented view of the fetal environment. How, as the social anthropologist Megan Warin has written of Barker’s hypothesis, was the “societal environment *telescoped* into the bodily environment of the womb” (Warin et al., 2012, p. 458, our emphasis)?

Our essay aims to answer this question by focusing primarily on the British context, as this is where the fetal origins hypothesis first gained traction, before it was exported to the rest of the world in the 1990s. For all the association of DOHaD with David Barker, we argue, the field itself is a hybrid of the two understandings of the developmental environment, which came together for the first time at the 1989 conference (Hanson, 2015). One came from fetal physiology and neonatology, with its close focus on the organism as it developed and matured, transitioning the threshold of the birth and gaining the ability to function outside the uterus. The other, while ostensibly coming from epidemiology, harked back to much older disciplinary settings of social medicine, demography, and statistics, where the fetus was not an endpoint in itself but rather an indicator of the state of the environment as well as a stage in the production of a healthy population. In this essay, we start from the Villa Marigola meeting to then look closely into the two divergent concepts of environment as they had developed in decades before 1989. We then return to the meeting and its aftermath, to discuss the losses and gains produced when these concepts came together and were promoted as the basis for a new paradigm in public health and health policymaking.

## 1989, Fetal autonomy and adaptation

The Villa Marigola meeting was named *Fetal autonomy and adaptation* to remind the participants of the significant meeting of fetal physiologists that took place in 1968 in London, titled *Foetal autonomy* (Wolstenholme & O’Connor, 1969). Opening the 1989 conference, Geoffrey Dawes described the 1968 meeting as an important transitional moment because “evidence had been accumulating that the [fetus], far from being a feeble diminutive version of the adult, was well able to regulate its own sophisticated affairs” (Dawes, 1990).

As we will show in the next section, the period between 1960 and 1980s was a productive time for research questions that foregrounded fetal autonomy. Yet by 1989, as Dawes indicated, concerns had accumulated that there was something missing. At the meeting fetal physiologists ascribed this shift in their thinking to scientific advancements, especially new methods in fetal research that allowed experimental observation on live, unanaesthetised fetuses. “We’ve been used to regarding the intrauterine environment as a quiet, soft and silent place, protected and free of stress,” wrote the obstetrician Alberto Zacutti, the Italian host of the Marigola meeting, in his introductory contribution (Zacutti, 1990). “This static condition, which was considered ideal for the fetus, its autonomy and homeostasis, is well represented in Leonardo’s famous picture, which reflects very accurately the anatomical character of the fetus pictured in a quiet curled up posture.”

**Fig. 1:**
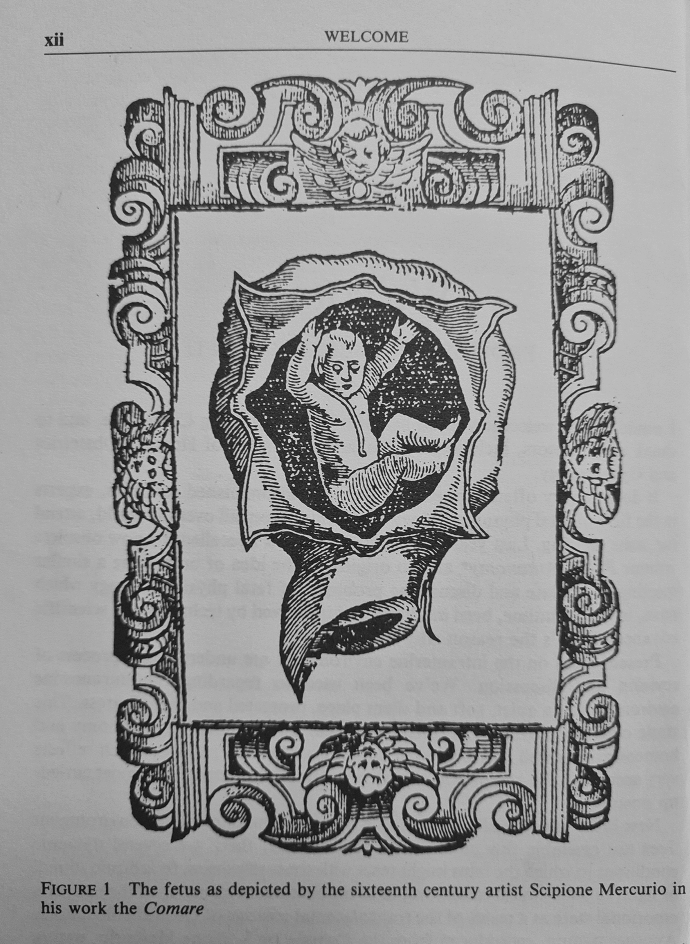
“Fetus” in Comare. Zacutti describes the image as “the fetus as depicted by the sixteenth century artist Scipione Mercurio in his work the Comare”. The reference appears to refer to Girolamo Mercurio, *La commare o raccoglitrice dell’eccellentissimo signor Scipion Mercurio: Divisa in tre libri*, Verona: Francesco de’ Rossi, 1642. From Zacutti (1990, p. xii)

This image of a peaceful, protected embryo stood in contrast to newer conceptions of a responsive organism in a dynamic environment. “New ideas are spreading, according to which the intrauterine environment does not resemble this picture,” continued Zacutti. “On the contrary, there are several dynamic conditions to which the fetus might react with stress responses, to acoustic stimuli or light, to biochemical conditions, or to the continuous changing of the mother’s emotional state as a result of the transplacental passage of chemical mediators.” Zacutti illustrated the transition using an image from a seventeenth century Italian midwifery manual (Fig. 1 Fetus in Comare).^1^ Typical for the period and the genre, it showed a fully formed, bouncy child, rather than a developing form (Whiteley, 2019).

Zacutti intended to contrast the static with the dynamic, indifference with responsiveness. But the choice of image ironically contradicted the other part of Zacutti’s, and Dawes’ argument: their warning that the fetus should not be seen as a small version of the adult. As stated by Mark Hanson, the British fetal physiologist who authored the chapter on “Fetal baroreflexes and chemoreflexes: Autonomy and adaptation” in the conference proceedings, *adaptation* mentioned in the title referred to the “degree of innate suitability to the environment”. It was distinct from *acclimatization*, which referred to** “**the changes that the organism may undergo, if its environment changed” (Hanson, 1990 p. 5).^2^ The job of the fetal physiologists was to study fetal functions not in isolation but in relation to the environment. Setting a successful research agenda for the field, then, hinged on recognizing how to conceptualize and quantify the fetal environment.

Chapters in the proceedings of *Fetal autonomy and adaptation* show how conference participants conceptualized the fetal environment and evaluated fetal responses to environmental factors. Physiologists imagined the environment as a network of measurable signals of physical and chemical nature: pressure, sound, vibration; oxygen, nutrients, and hormones. A change in one or more would trigger a cascade of responses. For example, a brief decline in blood oxygen would cause a decrease in heart rate in the short term. Yet if low oxygen lasted longer, it would prompt prioritization of blood supply to key organs and functions – brain and heart in the first place. This prioritization would in turn lead first to reduced movement, and then reduced growth of the limbs. It would, eventually, lead to an increase in the hormone erythropoietin that stimulated red blood cell production. And if all attempts at adaptation failed, the fetus could, ultimately, escape the hostile environment** “**by initiating birth”, mused Dawes (1990, p. 3), a claim reminiscent of earlier work on fetal autonomy. But the limits of fetal adaptation remained unclear, as well as its consequences.

Bringing Barker to a fetal physiological meeting was a provocation, as Dawes knew very well:

I look forward to our tackling this issue from a multidisciplinary point of view using the knowledge of traditional fetal physiology and endocrinology, supplemented by the skills of the molecular biologist and embryologist. I also wonder how our obstetric and neonatological colleagues will adapt to the problems which must arise from such an epidemiological surprise. (Dawes, 1990, p. 4)

Yet Dawes also recognized a potential alliance, and the enticing prospect of “a new stimulus to future research” (ibid.). David Barker was invited on the back of the work undertaken with the statistician Clive Osmond and other collaborators at his MRC Environmental Epidemiology Unit. Their key argument was that the increased morbidity and mortality from cardiovascular disease–the leading cause of death at the time—was caused by the deprivations of the early decades of the twentieth century, when contemporary heart disease sufferers were children.

This hypothesis went against the grain of mid-century epidemiological studies, most famously the work of Ancel Keys and the Framingham Heart Study. This research argued that adult lifestyles, namely smoking and food rich in saturated fats were “risk factors” for cardiovascular disease incidence (LaBerge, 2008; Rothstein, 2003; Tracy, 2019). Barker also used different methods. While the Framingham Heart Study relied on prospective epidemiological studies in adult cohorts, Barker combined contemporary disease statistics with both indirect and direct evidence of infant deprivation. The latter required a historical approach and archival data.

**Fig. 2:**
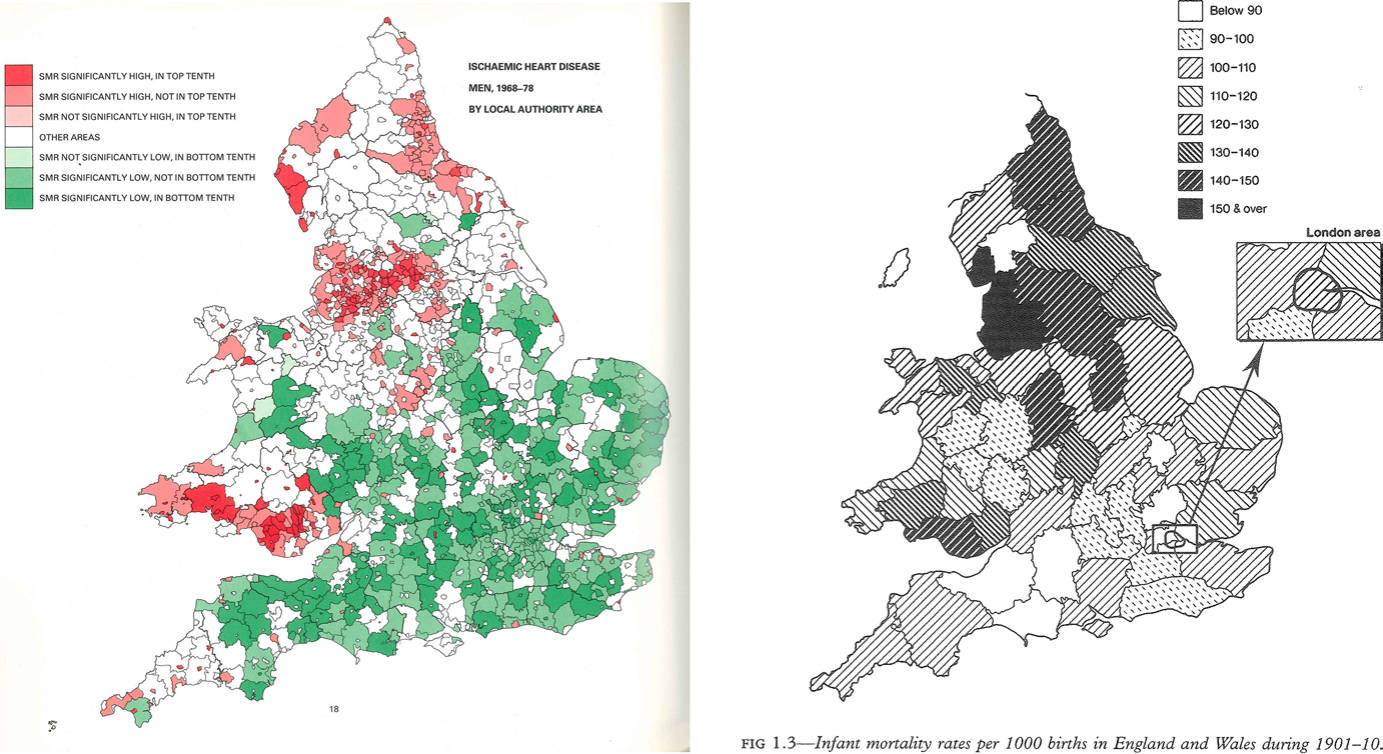
Barker’s hypothesis in maps. The left map shows standardized mortality ratios (SMR) of ischaemic heart disease for men in England and Wales, 1968–1978. Red-coloured counties depict areas indicate counties with “significantly high” or “high” SMR, while green-coloured counties are those with the SMR “significantly low” or “low” (Gardner, Winter, Barker, p. 18). The right map, published in Barker (1994, p. 4) but based on a map included in the Registrar General’s Statistical Review of England and Wales, 1901–1910, shows the infant mortality in the early twentieth century. These maps were often paired, to communicate the geographical link between the tough living conditions in the early twentieth century and the (cardiovascular) mortality risk decades later

Barker began by correlating the data on cardiovascular morbidity in England and Wales between 1968 and 1978 with data on infant and neonatal mortality some 60 years earlier (Gardener et al., 1984; Barker & Osmond, 1986) (Fig. 2 Barker’s hypothesis in maps). He found a geographical correlation between high cardiovascular mortality (in men) and infant mortality; and then hypothesized that the causal link was poor early life nutrition, which in turn was caused by maternal malnutrition and ill health, infant infection, and practices of artificial feeding.

These “ecological” (Barker’s term) findings were then supplemented by cohort studies on a group of men born in Hertfordshire around 1920, whose weights in infancy were (unusually) recorded and records preserved (Barker et al., 1989a, 1989b). Adult mortality records showed that those born very small–and especially those who then failed to ‘catch up’ in the first year of life, possibly because the life conditions of their families remained difficult–had a much higher relative risk of death from cardiovascular disease. Further cohort studies showed that people (but especially men) born small had higher (systolic) blood pressure in adulthood, a risk factor for heart disease.

Finally, the analysis of data from Preston, an industrial town in Lancashire, in the north of England where the local maternity hospital had kept detailed records for every woman admitted for decades allowed Barker to construct a tangible, material link between the maternal and the infant body. Namely, the records contained not only the infant weight and length but also the weight of the placenta. The information then allowed Barker to track 139 men and 120 women born in Preston between 1935 and 1940, still living near Preston, visit them, and establish a positive link between the placental weight and systolic pressure in adulthood (Barker et al., 1990).

Barker presented a summary of his work to physiologists in a short paper entitled “The intrauterine origins of adult hypertension” (Barker, 1990). He did not speak the language of fetal physiologists: he mentioned no chemoreceptors or baroreceptors, pituitary hormones or oxygen concentration. Yet he clearly wanted to make his work relevant to experimental scientists, in order to persuade them that the distant world of early twentieth-century industrial towns and rural communities could be distilled into tractable research hypotheses. In fact, even before the Marigola conference his papers show a gradual narrowing of focus and a methodological shift: from ecological studies to cohorts and then from cohorts indicating linkages between environment and disease aetiology, to the examination of placentas. In a 1981 paper on geographical variation in disease, Barker had written of aetiology as a “web of interrelated influences” that only “interaction [between epidemiology and] … ideas generated in clinical practice and the laboratory” could unravel (Barker, 1981). His 1986 paper talked about “poor living conditions” and discussed the effects of poverty (being born to a blue-collar family) upon infant mortality and, relatedly, later life health (Barker & Osmond, 1986). In 1989, by contrast, he referred to the “intrauterine environment” (Barker, 1990, p. 29). Yet although placental weight was meant to stand both for the mother and the world beyond her, in this reductionist move, much was lost.

In the discussion following Barker’s paper, recorded in the conference proceedings, attendees probed for weaknesses in the argument and suggested alternative, or supplementary, factors that could explain the patterns that Barker found. They proposed maternal smoking, “real” prematurity (meaning the fetuses that were born early rather than appearing premature because of their small size), genetic factors or social class. But it was the studies of the placenta that really spurred their imagination. Fetal physiologists could see how they could improve on the crude measure (of weight) that Barker used. What is surprising, however, is not that this audience were interested in Barker’s placental studies, but that they had not studied this question earlier. A quote in the discussion after Barker’s presentation at Villa Marigola reveals how fetal physiologists’ close focus on the fetus excluded even the closest maternal tissues:

Dawes wondered what was known of the control of placental growth. This seemed to be a neglected subject. Many meetings and reviews had been concerned with the determinants of fetal growth, but few had thought it worthwhile considering the problem of placental growth. (Barker, 1990, p. 37)

To understand how and why physiologists excluded the placenta from their programme, and why they practiced such a narrow view of the fetus, in the next section we go back a few decades to the beginnings of fetal physiology as a research field.

## Physiological approaches to the fetal environment

Scientists and historians of science today usually locate the beginnings of fetal physiology—as a well-defined field with its research questions, methodology and animals—in the 1930s Cambridge, when Joseph Barcroft, after a successful career in adult respiratory and cardiovascular physiology, took his interest in oxygen absorption from adult to fetal bodies (Giussani et al., 2016; Longo, 2016; Buklijas, 2014).^3^ Barcroft died in 1947, shortly after the publication of the foundational text of this new field, *Researches on pre-natal life*. Here he set out the broad programme of fetal physiology, which included the growth, in weight and length, and the development of organs and organs systems, as their form and function changed towards birth (Barcroft, 1947). Alongside these questions, Barcroft and his collaborators and assistants, many of whom later took this research programme to other institutions and countries, developed methods to study fetal physiology.

The study of animal fetuses, protected from direct observation and manipulation by their uterine enclosure, was far more difficult than the study of adult organisms. To conduct any measurement or experimentation on the fetus, fetal physiologists had to open the uterus of a pregnant animal and, leaving the placenta and cord intact, place the fetus in a water bath in which it could be observed visually or using electrodes (Barcroft, 1947; Huggett, 1927). But the fetus could not survive long like this: this procedure, usually referred to as “acute preparation”, allowed the observation of acute responses rather than long-term (“chronic”) monitoring of fetal development and behaviour. Whether results obtained in these “acute” studies could be extrapolated to the fetus-in-utero, in un-anaesthetised mothers, was not clear.

“Acute preparations” were complemented by indirect methodologies. At Cambridge’s Department of Experimental Medicine, the physiologist Robert McCance (1898–1993) and nutrition scientist Elsie Widdowson developed a research programme that began with fortifying wartime bread loaves and continued with the study of the effects of undernutrition during the economic crisis of the 1930s and then wartime deprivations (Buklijas, 2014). In 1946, McCance and Widdowson travelled to war-ravaged Germany on MRC funding, to study the impact of famine on populations as diverse as adult men, children in orphanages, and nursing mothers and infants. Upon their return to Cambridge, they turned observations collected on human participants into animal experiments. These experiments tested, on a number of animal species, how dietary changes through prenatal and very early postnatal life influenced later life health.

This research programme focused on the composition of experimental animal diets and the timing of nutritional variation, in order to understand whether there are “sensitive periods” when the organism or specific organs are especially sensitive to nutritional manipulation. McCance separated the role of the maternal organism as the nutritional environment from external (environmental) elements such as food access. “Until such time as we have perfected an artificial placenta, it will not be possible to investigate the food intake of a [fetus] directly,” wrote McCance (1962, p. 671), as he embarked on an artificial placenta project with his collaborator Lawrence Lawn, at the University of Cambridge’s Department of Experimental Medicine (Lawn & McCance, 1961). They framed this project as a clinical therapeutic device, supporting the development of prematurely born infants until their lungs matured, but the device was too complex and simpler and cheaper treatments for infant prematurity were developed (Buklijas, 2014). While ostensibly technical obstacles led to the abandonment of the artificial placenta project in the early 1970s, there was likely another reason for the decline in interest: the substitution of the “acute preparation” with the “chronic preparation” in fetal research (Guisanni et al., 2016; Meschia et al., 1965; Rudolph & Heymann, 1967). Instead of delivering the fetus into the water bath, in the “chronic” model indwelling catheters were placed into uterine arteries of the pregnant animal, usually sheep, to measure blood gases and pH. Once these catheters were inserted, the uterus was closed, and the animal was permitted to recover from anaesthesia. Indwelling catheters then allowed sampling of the fetal blood and further, longer-term observations.

The introduction of the “chronic preparation” was an upgrade on “acute” approaches: instead of the premature delivery for a short period of observation, the fetus could now complete its development under close monitoring. The pregnant animal and the fetus could recover, and the study could be claimed to be free from confounding effects of pre-operative fasting, anaesthesia and surgical stress. Yet it can be argued that what it did, primarily, was enable a strict focus on the fetus, while setting the environment aside.

This is not to say that technology alone brought about the concept of the autonomous fetus. Rather, the growing focus on the fetus in this period spurred investment into technologies that could address the questions that the physiologists wanted to ask. One window into the fetal physiologists’ perceptions of the fetus and its relationship with the environment are the metaphors that they used. McCance described the fetus as a “weightless astronaut in utero” (Jonxis et al., 1964, p. 307) while other fetal physiologists used metaphors borrowed from space and extreme altitude exploration and sports, comparing the uterine environment to “Everest in utero”—a metaphor first proposed by Barcroft (Giussani et al., 2016, p. 1107)—and the fetus to “diver” and “climber” or “mountaineer” (Dawes, 1968). They all invoked images of breathless effort in the face of “extreme” environments (Heggie, 2019), yet conditions of low oxygen and low barometric pressure were not difficult for the fetus: in fact, the fetus was adapted to them.

This shift in fetal physiology was closely related to the increasing focus on the fetus in medicine and in popular culture driven by demographic and social changes post- World War Two, as well as medical technology (Casper, 1998; Dubow, 2011; Jülich, 2015; Löwy, 2014). Antibiotics and vaccines changed the landscape of infant mortality, with infant death becoming a much rarer and more visible event, further foregrounded by the pronatalism of the Baby Boom era and broadened access to antenatal care and hospitalized childbirth (Marsh & Ronner, 1996, pp. 183–185; Al-Gailani, 2018; Golden, 2018). Ian Donald’s invention of the fetal ultrasound in the 1950s made the fetal body visible with less risk than earlier x-rays (Nicolson & Fleming, 2013). In New Zealand, William Liley conceptualized the fetus as a patient in its own right when he performed the first explicitly fetal treatment, administering blood transfusions through the uterine wall, into the bellies of fetuses suffering from Rhesus factor disease (Casper, 1998).

Many, especially in the West, learnt about human development from the photography of the Swedish reporter Lennart Nilsson (Jülich, 2018). Published as a photo-essay in *Life* magazine in 1965 and then in an illustrated book translated into many languages, Nilsson’s fetal photographs have been used towards many and sometimes opposing purposes: to educate future parents; in sex education; but also as arguments for fetal humanity, made by the organized anti-abortion or “pro-life” movement which grew markedly in the United States in the aftermath of Roe v Wade (1973) and, later, in Britain amid efforts to challenge the Abortion Act of 1967 (Dee, 2019; Sheldon et al., 2022). These photographs were also criticized for their close focus on the fetus and erasure of the mother. For that reason, they (and their author) were seen as complicit in the reactionary response to the feminist movement (Katz Rothman, 1986; Sofia, 1984; Pollack Petchesky, 1987). But the broader cultural impact of these photographs was considerable. One of Nilsson’s best-known images shows the “astronaut” fetus floating in its capsule; “Starchild” in Stanley Kubrick’s 1968 film *2001: Odyssey in Space* directly drew on this iconography. Such representations of the fetus as an astronaut in space played on the contemporary fascination with space exploration, culminating with the 1969 moon landing. Yet while the making and uses of pictorial representations of the autonomous fetus both in popular culture and in clinical medicine have received considerable attention (Morgan, 2009), the role of this concept in experimental research remained unexamined (Schoen, 2015).

The year 1968 could be regarded as the heyday of “classical” post-war fetal physiology. By then the “mantle” of the field leader had transferred from Cambridge to Oxford and the Nuffield Institute for Medical Research and Geoffrey Dawes, who became its first director only a few years after obtaining his medical qualifications. In 1959 Dawes co-founded the Neonatal Society, a forum that brought together experimentalists and clinicians with shared interests in the fetus and its transition to extrauterine life (McCance, 1968). This interdisciplinarity would remain the core feature of fetal and neonatal physiology. Also in 1968, Dawes published his influential book on *Fetal and neonatal physiology*. Perhaps more significantly, a meeting to contemplate the conceptual foundations and future research questions of the field took place in London, under the auspices of the Ciba Foundation.

The Ciba Foundation was a charitable organization established by the Swiss pharmaceutical company Ciba in London in 1948 to foster international scientific cooperation (Pickering, 1978). The Foundation’s main activity included small residential symposia held in its own “splendid” premises in London’s Portland Place. Described as a “scientific impresario”, the Foundation was much more than a funder. Under its director Gordon Wolstenholme it shaped the focus and format of meetings, which centred on cutting-edge biomedical questions and novel fields of inquiry: transplantation, genetics, developmental biology, cancer research and immunology. Wolstenholme and the Foundation had been involved in supporting fetal and neonatal research from the early days: in fact, the first meeting of the Neonatal Society in 1959 took place in Ciba’s London premises. But what is more interesting was the specific focus on *Fetal autonomy* as indicated by the title (chosen by Dawes and Wolstenholme together). In the introduction to the conference proceedings, Dawes argued that *“*[the fetus] demonstrates its innate capacity for influencing its external and maintaining its internal environment–that is, its autonomy” (Wolstenholme & O’Connor, 1969, p. 1).

The volume contained 15 papers on a host of topics including cardiovascular development and function; fetal metabolism and weight gain; neuromuscular development; hormones in programming the sexual differentiation. Titles such as “The extent of foetal endocrine autonomy” or “Independently regulated synthetic transitions in foetal tissues” indicate the focus on the fetus as an autonomous organism, but the overall argument went further than that. Take the example of the New Zealand physiologist and obstetrician Graham “Mont” Liggins whose interest in the causes of premature birth led him to the question of the control of labour in the preferred pregnancy model: sheep (Gluckman & Buklijas, 2013). His PhD dissertation at the University of Auckland confirmed the hypothesis that the fetal lamb makes an essential contribution to the initiation of birth, through the hormones released by the fetal adrenal cortex controlled by the fetal pituitary gland, in turn governed by the fetal brain (Liggins, 1969). Liggins’ work was much admired and later led to important clinical advancements; yet the origins of the question in the notion of the autonomous fetus has not been appreciated.

The 1968 meeting was followed by further Ciba Foundation conferences about *Size at Birth* (1974), *The Fetus and the Birth* (1977), rounding off the series in 1981 with a meeting on *The Fetus and Independent Life* (Elliott & Knight, 1974; Knight & O’Connor, 1977; Elliott & Whelan, 1981). Contributions to these volumes depicted the fetus in the uterus as a closed system: and although many among the contributors studied change, they focused on transformation across time, as the organism approached the moment of birth, rather than changes caused by environmental modifications.

For British scientists, fetal research had gained new political charge as “pro-life” opponents of abortion liberalization grew in prominence in the later 1970s and ‘80s. Campaigns to reduce the upper time limit of legal abortion, within and beyond parliament, increasingly drew on scientific, medical and technical understandings of fetal development and viability to appeal more widely to a secular audience (Franklin, 2014; Franklin et al., 1991). Though always smaller in profile and less legislatively successful than US counterparts, anti-abortion efforts in Britain made similar use visual arguments, including the now routine technology of obstetric ultrasound, to present a morally infused vision of medical science activists claimed to be revealing new truths about “humanity” of the unborn (Sheldon et al., 2022). Similar historical developments can be seen in other European countries where access to safe abortion, legalized in the previous decades, came under attack from the late 1980s onwards. Dagmar Herzog has argued that in Germany this backlash was prompted especially by more positive attitudes towards people with disabilities on the back of the post-war generation’s rejection of the Nazi past and the legacies of eugenics (Herzog, 2018). In much of Eastern Europe liberal abortion legislation was seen as entwined with the socialist culture that these countries were now disestablishing. Italy, where the Villa Marigola conference took place, saw a similar counteraction in the years after the 1978 legalization of abortion (Haberman, 1989). While we do not know much about the politics of the conference’s host, Alberto Zacutti, as a pioneer of obstetric ultrasound in his country and a senior obstetrician, he was likely drawn into these debates.

At the same time, fetal physiology was under pressure from the inside too. New molecular methodologies and disciplines–biotechnology, reproductive technologies, developmental biology and genetics–were all competing for research funding amidst political and institutional pressure to cut costs and modernize science (Garcia-Sancho, 2015; Myelnikov, 2017). For fetal physiology, this meant proving its relevance to questions of broader importance–namely, adult health–through a change in focus and building new constituencies with clinicians and medical scientists (Hanson & Buklijas, 2024).

At the time of the Villa Marigola meeting, then, the field had been working with the same set of concepts, research questions, and methodologies for several decades that had been consolidated by the Ciba meetings. But the social, political environment had changed, as research on fetal autonomy fed into arguments over viability and abortion. New disciplines and methodologies vied with physiology for increasingly tight research funding. These two factors–the field lacking novel ideas and the changed socioeconomic environment–explain Dawes’ eagerness to step away from the core paradigm of the “autonomous fetus” and recruit fresh approaches. In the next section, we turn to David Barker and the intellectual traditions to which he belonged, considering the role that the fetal environment had in their research programmes and public health objectives.

## Social medicine and post-war congenital malformations epidemiology

Barker came to the Villa Marigola meeting with an understanding of the fetal environment that was quite different from that of the physiologists. His view was rooted in a significant part in the epidemiological research tradition of social medicine that took shape in Britain following World War Two. Social medicine provided the main disciplinary context for new ways of conceptualizing the relationship between fetus and environment that began to emerge from the 1940s, and which Barker would revisit in the early 1980s. In the immediate term, these approaches helped consolidate medical and public health interest in the ‘perinatal’ period as a focus of research and clinical intervention, concentrating on the fetus and newborn (Weir, 2006). Institutional and intellectual arrangements in post-war Britain fostered particular interests in the social epidemiology of reproduction. The “steady output of epidemiological studies dealing with complications of pregnancy and particularly with perinatal death”, such that medical sociologist Raymond Illsley could remark in 1966 that “in no other period, and no other country, has perinatal mortality been better recorded” (Illsley, 1966, p. 181).^4^

Late to develop in Britain compared to most other European countries, the formal discipline of social medicine grew out interwar interest in the social aspects of health and disease, in the work of Richard Titmuss and John Ryle, among others (Oakley, 1991, 2019).^5^ But significant support for social medicine emerged only in the 1940s amid a general surge in interest in social issues and support for redistributive social policies. The field gained impetus from the rapid growth of occupational health research amid a desire to improve conditions in the factories whose production was an essential element of the war effort. Four chairs in social medicine at Oxford, Edinburgh, Birmingham and Sheffield, and a new Institute of Social Medicine at Oxford gave the discipline an institutional foothold, then in 1948 the UK Medical Research Council established a Social Medicine Research Unit, headed by epidemiologist Jerry Morris. Although the objectives of these departments were always indicative and sketchy, a key motivation for research was establishing a clearer link between health service provision and population need (Murphy, 1999; Porter, 1997). A broader political context linking medical research with support for redistributive social policies, the growth of the postwar welfare state and the shift from the Empire to the postcolonial nation-state–1948 was also the year of the introduction of the British Nationality Act and of the founding of the NHS–is only beginning to be understood (Bivins, 2015; Moore, 2016).

This progressive, humanistic and “needs driven” epidemiological orientation chimed with the political focus on poverty that characterized the original analytical framework of social medicine. Analysing the relationship between socio-economic inequality and differential distribution of health and disease was a foundational agenda of the discipline. As Dorothy Porter has argued, this group shared a strong commitment to the analytical power of quantitative methods and the potential political power of epidemiological research to influence social change through policymaking. For this generation of academics, social medicine and a needs-driven epidemiology were more or less interchangeable descriptions of the quantitative, population-based scientific investigation of disease, health, and socio-economic inequality aimed at instituting social change. Porter has termed this fusion of quantitative empirical analysis to the institutionalisation of social change the “political positivism” of Britain’s first generation of social medicine academics. The epidemiological link between inequality and disease causation supplied social medicine with its scientific authority and disciplinary identity as well as its political rationale (Porter, 1996, 1997).

This identity was promoted, above all, by Barker’s PhD supervisor Thomas McKeown, who had been made professor of social medicine at Birmingham University in 1946. McKeown was a disciple of Lancelot Hogben who, together with Francis Crew, was wedded to statistical measurement as the basis of scientific validity within the social and biological sciences, and was the earliest and most influential exponent of the view that heredity and the environment were interdependent (Tabery, 2014). Much of the work coming out of McKeown’s department at Birmingham in the decade or so after it was established concentrated on the epidemiology of congenital malformations. This research programme followed from a larger interest in what was termed “reproductive efficiency”, which encompassed the relationship between human reproduction, social conditions and physical and mental health. This line of work was pursued elsewhere, too. Richard Doll’s and Austin Bradford Hill’s statistical demonstration in 1958 of the causal relationship between maternal rubella and congenital defects, like their more celebrated studies of smoking and lung cancer, came to symbolize the promise of the quantitative methodology of post-war social medicine (Hill et al., 1958). Morris’ new MRC unit, meanwhile, collaborated with the General Register Office to produce a then ground-breaking prospective study of stillbirth and infant deaths in England and Wales during 1949 and 1950, exploring the influence of such factors as the mother’s age, her parity, social class and geographic region, as well as the father’s occupation, on mortality rates (Morris & Heady, 1955).

The MRC also funded the Aberdeen obstetrician Dugald Baird and his research on social aspects of reproductive health. Strongly supportive of women’s access to contraception and legal abortion, Baird produced pioneering studies providing evidence of the social and economic factors in stillbirth and poor maternal health, which he recognized as rooted in the deprived conditions he observed in Aberdeen (Baird, 1945). In 1948, Baird set up the Aberdeen Maternal and Neonatal Databank, which has since its inception collected research-standard data on the pregnancy and birth outcomes of at least 85% of women delivering their babies in the city. This work, considered a model of how the approach of social medicine could be applied to a clinical problem, led to the establishment in 1955 of an autonomous Obstetric Research Unit (Bhatia, 1996; van Teijlingen & Barbour, 1996; Davis & Davidson, 2006).

McKeown’s department at Birmingham, however, was in the postwar years the focal point of quantitative research on human reproduction within the social medicine tradition. This was sustained by a longstanding interest in prenatal development—McKeown had begun his academic career in Montreal researching the physiology of the mammalian placenta (Selye & McKeown, 1935)—and the mentorship of Hogben, who had a new chair of medical statistics created for him at Birmingham in 1947. On taking up his own professorship of social medicine in 1946, McKeown initiated a programme of research on the incidence of congenital malformations that would occupy him, and colleagues at Birmingham, for the following two decades. Hogben oversaw the early publications to come out of this programme, in which McKeown and his main collaborator and “right-hand-man”, the obstetrically-trained epidemiologist Reginald Record, set out the motivation for the research by invoking their mentor and his 1933 book *Nature and Nurture* (Leck, 1996). Highlighting the limitations of laboratory research on standardized animal stocks in carefully controlled conditions, the authors argued that an epidemiological approach to congenital malformations would help test Hogben’s hypothesis about the “interdependence of genetic and environmental variables”. For McKeown and Record, this was no mere “academic exercise”, but offered the key to understanding and developing a preventative approach to what seemed to many epidemiologists at this time a new and significant frontier in public health (Record & McKeown, 1949, 1950a, 1950b).

This view emerged from, but also helped to shape, the post-war consensus that the “epidemiological transition” from predominantly infectious to predominantly chronic diseases in Western societies provided new problems for quantitative socio-medical analysis to elucidate, such as the causation of human malformations. The decline in mortality from other disorders of early life had led to an increase in the proportion of early deaths caused by congenital malformations; moreover, with more children born with mental and physical disabilities surviving beyond early infancy, parents, medical practitioners, public health officials and service providers were beginning to articulate the urgency of state support within the framework of the post-war welfare state (McKeown, 1976; Weatherall & Haskey, 1976). Meanwhile, recent evidence showing that malformations could result from maternal rubella and high doses of ionising radiation provided incentives to apply the tools of social medicine to analyse the relative importance of inheritance and the environment. These factors made malformations an enticing research problem, with practical as well as theoretical implications, for a “needs driven” epidemiology.^6^

In McKeown’s and his colleagues’ epidemiology of congenital malformations, understanding the “fetal environment” meant developing ways of analysing such variables as year, season and place of birth, birth order, mother’s age and social circumstances. Long recognized as significant for infant mortality, these variables were by the 1950s more broadly linked to perinatal health, including congenital malformations. The Birmingham approach would turn above all on access to data through a close and longstanding partnership with the local health department. Particularly important was the enthusiasm and support of officials in charge of maternal and child welfare and the city’s Central Statistical Office. These municipal health workers shared the ethos of social medicine, recognized the value of epidemiological research for benefiting mothers and children, and saw it as their responsibility to share records freely with McKeown’s department, including by facilitating interviews with affected families and access to local maternity services. Most innovative were the birth registers, set up with Hogben’s guidance in 1950, which documented clinical and demographic data from maternity hospitals, domiciliary midwives and health visitors, as well as stillbirth and death certificates and autopsy reports (Edwards et al., 1964; Record & McKeown, 1950a, 1950b). This led to the first population-based register of malformations to be initiated as an ongoing programme. The Birmingham register, linked through the birth register to extensive parental and personal data for the related population, allowed for the first time “cohort” studies of human malformations, establishing a dataset on which subsequent research would draw (Leck, 1996).

If McKeown’s programme provided a methodological framework for congenital malformations epidemiology, in the short term it crystallized British researchers’ interest in malformations of the central nervous system (CNS) such as spina bifida, anencephaly and congenital hydrocephalus. More systematic approaches to registering malformations confirmed that these were relatively common, and evidently unusually so in the British Isles compared to many other countries. This made CNS malformations an ideal candidate for investigating the influence of environmental variables, including seasonal and secular variations, geography, and especially social class effects in the wider context of the “rediscovery of poverty” and recognition of the intractability of inequality in the 1960s (Lowe, 1995; Webster, 2002). As Baird would later put it, CNS malformations “proved to be a very useful tool for studying, on a national scale, the relation between socioeconomic conditions and reproductive efficiency” (Baird, 1985). More immediately, this work encouraged health researchers and health workers to view such malformations as “one of our more urgent medical, social, epidemiological and aetiological problems” (Laurence & David, 1964; Laurence et al., 1968; Nevin et al., 1981).

Initially oriented toward the study of social class effects, congenital malformations epidemiology took on new salience and scope after 1961, as medical practitioners worldwide began to recognize the harmful effects of the anti-nausea drug thalidomide on the developing fetus (Daemmrich, 2002). Establishing environmental risks to the unborn child and the prevalence of disability in the community became more urgent public health goals, leading to the expansion of congenital malformations surveillance, through the scaling up of local registries such as those initiated in Birmingham.^7^ In the wake of the thalidomide tragedy, a national system of reporting and data collection was instituted for England and Wales in 1964, coordinated by the General Register Office (GRO), reconstituted as the Office of Population Censuses and Surveys in 1970 (Weatherall & Haskey, 1976). Josephine Weatherall, the medical statistician who designed the surveillance system for the GRO, went on to become the founding project leader (from 1978 to 1984) of the European Register of Congenital Anomalies and Twins (EUROCAT), then based in Leuven, Belgium. Initially conceived in 1974, EUROCAT was the first “Concerted Action Project” of the Medical Research Programme of the new European Community, intended to demonstrate the benefits of scientific cooperation and the development of a unified European approach to a shared public health problem (Weatherall, 1985). The main justification for the EUROCAT project is that the visibility of environmental risks depends on monitoring and data collection on an international scale.

Even as congenital malformations epidemiology was extended and institutionalized in national and international surveillance systems, the intellectual parameters of social medicine and its relationship to public health were changing. With its allegiance to a model of pure research, social medicine grew detached from the practice and personnel of post-war health provision, failing to gain an institutional foothold as an academic discipline beyond the 1960s. Porter has linked this failure to the weakening of traditional structural explanations of core public health concerns, which were superseded by “behaviourist” epidemiological models and a new emphasis on “lifestyle medicine” from around 1960. Since the nineteenth century, studies of infant mortality had prioritized economic inequality as the major cause of steep differential gradients according to class. Within research on reproduction, socio-behavioural approaches were increasingly visible as part of the larger shift in focus to the perinatal period (Porter, 2002). Socio-behavioural investigations began to explore other factors in the early 1950s within the new framework of perinatal epidemiology, opening up interest in and concern with the influence of such factors as smoking and obesity on fetal and neonatal health (Weir, 2006). This new argument claimed that *lifestyles*, involving unhealthy behaviours such as excessive food consumption and lack of exercise, created major risks rather than *life conditions* such as economic inequality (Morris, 1957).

## David Barker and the environments of early life

Barker’s early career coincided with the consolidation of the “lifestyle approach” as the hegemonic epidemiological paradigm. The connections between individual behaviour and health, especially chronic disease risk, would remain the dominant narrative of Anglo-American public health and health policy. A revived emphasis on disease prevention campaigns during the 1970s, catalysed by financial pressures on health services, coalesced with shifting ideas about the welfare state and its relationship to the role of the individual (Clark, 2020). However, new interest in the complex relations between health and social inequality around 1980 was disrupting the consensus. The problem of inequitable distribution of health resources, and the failure of the post-war welfare state to correct historic patterns of disadvantage, remained a persistent concern in British public health, not least following the prolonged economic crisis precipitated by the oil price increases of 1973 (Webster, 2002). But by the end of the decade, novel strands of research, in tandem with official statistics and broader societal critiques, had reignited debate about “transmitted deprivation” and social and geographic disparities in health (Blaxter, 1981; Clark, 2021; Macintyre, 1986; Rutter & Madge, 1976). Most significantly, the Black Report, published in controversial circumstances by the newly incumbent Conservative government in August 1980, was a pivotal moment in bringing the new concept of “health inequalities” into view as both an epidemiological and public concern (Macintyre, 1997).

This was the context in which Barker revisited interests cultivated at Birmingham under McKeown. Barker’s doctoral research, published in a series of papers between 1967 and 1969, part of a larger project undertaken with McKeown, Record and the geneticist John Hilton Edwards, had focused on prenatal influences on subnormal intelligence (Leck, 1996). Motivated by a longstanding concern to understand the relative contributions of genes and environment in measured intelligence which explicitly harked back to Hogben, these studies investigated obstetric influences, birth weight and duration of gestation, birth order and maternal age as possible variables. While the researchers could draw no firm connections between these factors and intelligence, these studies established a methodological framework for conceptualizing and investigating the role of early environmental influences in development and subsequent health (McKeown & Record, 1976). As for the wider programme of congenital malformations epidemiology, the research hinged on collaborations with local authority health departments as well as the creative use of medical records, including historic data, where available, and welfare and education officers’ interviews with parents, to generate and test hypotheses about prenatal influence (Barker, 1966a, 1966b). Above all, these studies allowed the researchers to assert the epidemiological significance of the prenatal social environment, and established the outlines of an intellectual agenda to which Barker would return a decade later.

Barker’s participation in this work was curtailed in 1969, however, when he was seconded to Makerere University in Kampala, Uganda, on an MRC grant to study *Mycobacterium ulcerans* infection (buruli ulcer). With his contract in Uganda cut short by Idi Amin’s 1971 coup d’état, he returned to England to take up a position in clinical epidemiology at the University of Southampton. A “canny sleuth”, Barker developed a broad programme of research seeking epidemiological ‘clues’ to aetiology and evidence of modifiable environmental influences in a wide range of conditions from gallstones to Alzheimer’s disease (Lampl, 2014). Alongside this research, Barker co-authored with Geoffrey Rose two introductory textbooks *Epidemiology in Medical Practice* (Barker & Rose, 1976) and *Epidemiology for the Uninitiated* (Barker & Rose, 1979), both of which went through five editions. Based on a series of short articles published in the *British Medical Journal*, the latter work allowed the authors to return to first principles, defining such broad questions as “what is epidemiology?” for a medical audience.

The collaboration with Rose placed Barker within an intellectual milieu that was at precisely this time rethinking the narrative of public health in Britain. Rose was one of the lead researchers on the longitudinal “Whitehall Study” of chronic disease among 18,043 male civil servants, which helped to shape a wider conversation about the class system and its relations to health. As Peder Clark has argued, the novelty of the study lay predominantly in the researchers’ attention to class as a determinant of disease beyond the dominant focus on inequities of access. Rather, the research helped to build an argument as to how the effects of inequality might be “literally embodied by the incidence of disease” (Clark, 2021). Rose, and particularly Michael Marmot, who joined the Whitehall study in 1976, would play key roles in formulating a critique of lifestyle public health, which they argued failed to address the core issues of an unequal society (Rose & Marmot, 1981). The first publications to come out of the study, followed shortly by the Black Report, would stimulate an “outpouring of published material” on the social patterning of health as a “mood of self-criticism” took root in health services research during the global economic crisis of the later 1970s and as the politics of inequality became increasingly contested under the Thatcher government (Macintyre, 1997; Webster, 2002).

Barker’s return to the prenatal environment, the core agenda of his doctoral work with McKeown, then, took place in a context of renewed interest in and debate about the precise pathways by which social structure affects health. But by the early 1980s, Barker could also draw on new resources and strands of research that had not been available to him a decade earlier. In particular, Rose and Marmot’s reinterpretation of coronary heart disease in terms of class-based inequalities rather than a condition of affluence lay the ground for further work on social patterning of chronic disease (Macintyre, 1997). There was also more general concern with the causes and effects of “cycles of disadvantage” across generations. In 1972, Conservative health minister Sir Keith Joseph had raised the issue of “transmitted deprivation” across generations in the working classes and set up a seven-year government funded initiative to examine the reasons for the continuities of social deprivation in spite of welfare-state support. A succession of post-war longitudinal studies had linked social class and life chances, especially in relation to perinatal problems, education and child development, and mental health. By the 1970s, longitudinal data and cohort studies were being applied more broadly and more systematically as tools for investigating intergenerational and intragenerational patterns in health (Welshman, 2005, 2007). At the same time, there was a flowering of small area studies using composite indices of deprivation drawn from Census data, which compared differences in mortality and morbidity between more or less deprived wards (Carstairs, 1981).

At Southampton, Barker distilled those interests into a series of investigations into the importance of early life experiences to health. The earliest expression of this concern with early life influences came in a project on Paget’s disease (osteitis deformans), a cause of bone pain, fractures, and deformity in the elderly. In a 1974 paper with the statistician Martin Gardner, Barker posited that similarities between the geographical distributions of Paget’s disease in the elderly in the present day, and rickets in earlier generations, pointed to vitamin D deficiency in childhood as a predisposing cause (Barker & Gardner, 1974). These studies, which Barker continued to pursue into the 1990s, indicate the direction of this thinking on the complex relations between social change, deprivation and health across generations. Other projects led more directly from Barker’s early work with McKeown. Research on the geographical distribution of Perthes disease of the hip in children, then considered to be of genetic origin, led Barker to speculate about the still to be determined role of “prenatal influences”. With distribution maps revealing the condition to be more common in specific urban conurbations, Barker postulated that Perthes disease was a “late manifestation of a congenital disorder” with an environmental aetiology. By 1990, he was writing more conclusively about the “social origins” of Perthes disease on the basis of these studies (Barker et al., 1978; Hall et al., 1990).

By the mid-1980s, these suggestive correlations between early life conditions and later health had crystallized into a firm hypothesis about the fetal origins of adult disease. This stemmed directly from a mapping project, the *Atlas of Mortality from Selected Diseases in England and Wales, 1968–1978* (Gardner et al., 1984), which visualized the geographical correlation between the distributions of ischaemic heart disease mortality in the 1970s and infant mortality in the early twentieth century (Buklijas, 2018). Barker was now able to refer to a growing body of studies that were reporting similar effects. In the 1960s, South African epidemiologists Zena Stein and Mervyn Susser, then based in the United States, undertook a large programme of research on the reproductive effects of the Dutch famine of 1944–45, including on the relationship between prenatal nutrition and mental performance. This work highlighted the importance of timing or “critical periods” in nutrition and development (Smith & Susser, 2002; Stein et al., 1972). Most significantly for Barker’s work on chronic disease, Norwegian researcher Anders Forsdahl proposed that maternal living conditions during pregnancy and the first years of childhood were an important risk factor for arteriosclerotic heart disease (Forsdahl, 1977).

Barker’s interest in the long-term pathological effects of intra-uterine influences built on these investigations through the 1980s, in particular through his imaginative follow-ups of historical cohorts and by linking earlier research on “critical” or sensitive periods of development to adult chronic disease. The turn to ischaemic heart disease allowed Barker and his colleagues to apply the model, developed for relatively obscure conditions such as Paget’s and Perthes’ disease, to the central issue in social epidemiology in the 1980s. The landmark 1986 paper in the *Lancet* formally announcing the “hypothesis”’ participated in the broader reinterpretation of coronary heart disease, and specifically the weakening of its identity as a disease of affluence, and its associated challenge to the “lifestyle paradigm” in public health (Barker & Osmond, 1986). Echoing Marmot and Rose, the limitations of “known risk factors” as predictors of adult cardiovascular disease was a theme to which Barker would repeatedly return as he sought to promote the hypothesis as a cohesive programme of research (Barker & Martyn, 1992). More generally, as Barker would write in *Nature* in 1989 the approach required an entirely new model for understanding “Western diseases”, which replaced an emphasis on adult- with early life-environments (Barker, 1989). That even relatively early responses cast Barker’s research programme in terms of a “Kuhnian paradigm shift” was a measure of his success in marketing the approach in this way (Robinson, 1992). It also depended on the existence and consolidation of a “strong constituency” in the public health field who were receptive to such an interpretation (Kuh & Smith, 1993).

As debates around the pathways through which social structures shaped health intensified in the wake of the Black report, Barker’s hypothesis invited the prospect of an explanatory approach to the complex intergenerational effects of inequality and deprivation. It also, he claimed, promised to inform a “new national strategy for reducing inequalities of health in Britain”. “The seeds of inequalities in health in the next century are being sown today,” as Barker put it in an article published in the *Journal of Public Health Medicine* in 1991, “in inner cities and other communities where adverse influences impair the growth, nutrition and health of mothers and their infants” (Barker, 1991). This framing indicates the extent to which Barker’s hypothesis was rooted in a lively discussion of health inequalities, as epidemiologists and public health scientists redefined the type of critique they could make about the significance of social disparities. In the final section, we explain how efforts to promote the hypothesis as an explanatory framework and research agenda helped to coalesce new interest in and controversy around “fetal origins” and the relations between *intrauterine* environments and long-term health outcomes.

## Environment, mechanism and “fetal origins” research in the 1990s

For all the hyperbole around the fetal origins of disease in adulthood and its claims to herald a new model of public health, the hypothesis invited significant scepticism, notably among other epidemiologists who questioned both the strength of the available evidence and the novelty of Barker’s emphasis on early life influences (Kuh & Smith, 1993; Smith & Kuh, 1997). Researchers with long-term investments in cardiovascular epidemiology were the most openly critical, typically observing that Barker’s cohort follow-up studies dealt inadequately with the fact that the relation between adult cardiovascular risk and early life experience might be confounded by persisting social and economic disadvantage across the life course (Ben-Shlomo & Smith, 1991; Vågerö & Illsley, 1995). While accepting the “biological plausibility” of the fetal origins hypothesis, enthusiasts and critics alike demanded the as yet unavailable “experimental evidence” that would put the research on a more secure footing (Elford et al., 1991). For Barker, experimental connections, such as those he sought to cultivate at Villa Marigola, provided opportunities to test, and confirm, his hypothesis using the authoritative tools of laboratory science. What was gained, and what was lost, in this encounter between an epidemiological programme attempting to explain the complex intergenerational effects of inequality and deprivation and a firmly experimental and reductionist approach?

At Villa Marigola, fetal physiologists’ responses to Barker’s presentation ranged from cautiously positive to openly critical. The American physiologist Kent Thornburg said, for example, that it was “far-fetched that a short-term adaptation of a fetus in trouble would have such a long-term effect on the likelihood of ischaemic heart disease in adult life” (Zacutti, 1990, p. 36). Others worried that the effects of smoking or infections were not considered. Even so, “Barker stuck to his hypothesis” (p. 36). Yet within a few years, many of those present– including Thornburg—would become advocates for the new field. A follow-up Ciba meeting, convened by Barker in London in 1991, invited participants from child health, psychiatry, nutrition and neurology among other fields to “embark on a journey down many paths”, think creatively about the mechanisms which might explain early life influences on long-term health, and identify “concepts which unify research across the whole field” (Bock & Whelan, 1991, p. 2). As at Marigola, concerns remained, about the difficulty of disentangling early life influences from other variables, the collapsing together of “risk indicators” and “risk mechanisms”, the tendency of enthusiasts to oversimplify from historical data, and over how realistically still debated findings could be translated into policy. Among the most difficult objections to knock down was the charge of reductionism. Critics worried that Barker’s hypothesis failed to adequately take account of the “volatility of the environment” across the lifecourse, and how “developmental changes interact with multiple environmental changes, not simply a single environmental event” (p. 232).

Despite initial expressions of caution, fetal and neonatal physiologists for their part recognized they had much to gain from this alliance. Thornburg’s message to the 1991 symposium in London was that interdisciplinary research “linking the molecular basis of physiological processes and medical findings together with epidemiology” offered rich possibilities, and was in all participants’ interests (Bock & Whelan, 1991, p. 232). “Molecular” may have been the key word here: as discussed earlier, fetal physiology was a discipline at risk of losing support amidst funding cuts and growing political support for new “molecular” disciplines. The early years of fetal origins research were by no means easy and criticism as well as the absence of institutional support persisted through the 1990s. However, Dawes’ hopes of revitalizing his field through the experimental testing of Barker’s hypothesis were ultimately fulfilled beyond even his most optimistic expectations.

**Fig. 3:**
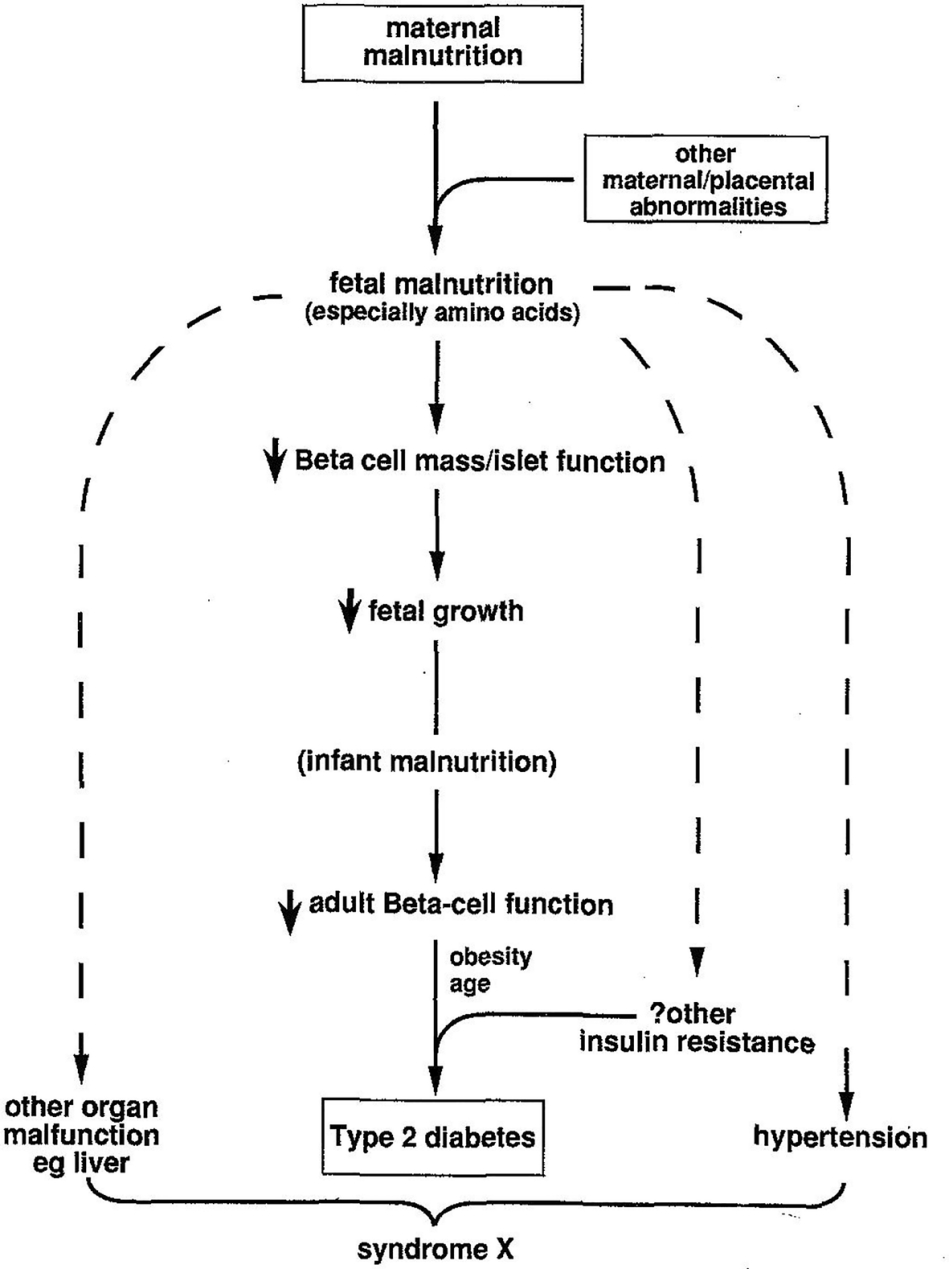
“Thrifty phenotype”. Diagrammatic representation of the “thrifty phenotype” hypothesis explaining the causation of “syndrome x” – a cluster of clinical conditions including hypertension and type 2 diabetes with aberration of lipid and insulin metabolism in the background. From Hales and Barker (1992)

Early collaborative publications started with reanalysing and reinterpreting existing animal and human data using the new framework. New concepts, namely “programming” and the “thrifty phenotype” were proposed in this period to describe the relationship between the developmental environment and the later life pathology. It is telling that these terms, meant to describe the fluid, complex links between the organism and the world around it were drawn from two fields seen as highly deterministic: computer science and genetics. “Programming”, usually ascribed to the Cambridge nutrition scientist Alan Lucas, stood for a process “whereby a stimulus or insult at a critical period of development has lasting or lifelong significance” (Lucas, 1991 p. 38).^8^ The notion of the “thrifty phenotype” riffed on the well-known if controversial proposition by the geneticist James Neel, who argued that the rising prevalence of metabolic disease (diabetes and obesity) today may be explained by the maladaptation of genes selected for an environment of nutritional scarcity to our calorie-rich world (Neel, 1962).^9^ Barker and the biochemist Nicholas Hales argued that “poor fetal and early post-natal nutrition imposes mechanisms of nutritional thrift upon the growing mechanism”. Investigation of mechanisms that could turn pathogenic should the organism find itself in a nutrition-rich environment (Hales & Barker, 1992: see Fig. 3) as well as the concept of “programming” would prove memorable ways to introduce a new field. Both of these terms would later come to be criticized from within the field: “programming” precisely for its determinism, and the “thrifty phenotype” because its full proposition was not supported by data (Gluckman et al., 2010). But they also proved extraordinarily productive, inviting intellectual debate and attracting researchers across a variety of disciplines to the field.

The encounter with the fetal physiologists, then, coincided with a broader shift in emphasis in the framing of fetal origins research as Barker came increasingly to highlight the search for “mechanisms operating in early life” that would support a model of disease which enjoyed only qualified support from epidemiologists (Bock & Whelan, 1991, p.1). As Barker’s work was becoming better known, criticisms of his foundational, “ecological” studies–seen as imprecise–and of the general argument regarding the relative importance of the early life environment and ongoing disadvantage, grew louder (Robinson, 1992). While part of the response to that scepticism involved extending prospective, case–control epidemiological studies, experimental work became ever more critical to showing the mechanistic link between early life events and physiological adaptation that would later manifest itself as a disease. Barker’s strategy for managing objections and maintaining the distinctiveness of his work hinged on the ability to step more fully into the world of the fetal physiologists and harness their approaches.

In the experimental, clinical and prospective epidemiological studies which proliferated through the 1990s and beyond, the rich intergenerational environments of inner cities and industrial towns made out of the airs, waters, workplaces and homes, nutrition, movement and relationships, were reduced to a simple, experimentally testable hypothesis. Studies would, for example, modify energy, fat or protein content of the feed given to pregnant animals and, sometimes, their offspring, and then observe a limited set of parameters associated with the risk of diabetes or cardiovascular disease (see e.g. Vickers et al., 2000). Towards the end of the twentieth century, researchers in developed societies identified obesity as the key issue of concern not only in the West but also and increasingly so in developing countries. Consequently, animal experimental as well as clinical and epidemiological research moved from the study of the effects of famine and undernutrition, to the study of the causes and long-term consequences of obesity, increasingly interpreted as resulting from mal- rather than overnutrition (Gluckman & Hanson, 2012). The perceived effects of early life malnutrition were often subtle, and the transferability of experimental conclusions from animals to humans was not always clear, in particular where, as social scientists began to argue, poor nutrition was only a symptom of broader problems in people’s lives that could not be remedied through targeted intervention (Egger & Swinburn, 1997; Warin et al., 2011, 2012). Yet fetal origins research nonetheless gained in popularity and influence, in particular in the policy space, including in the ongoing debate over health inequalities in Britain.

The policy implications of the intrauterine origins of cardiovascular disease had already been noted in a government white paper in 1992 (Department of Health, 1992, p. 117). But it was the Labour landslide of 1997, and the new government’s emphasis on tackling the “health gap” between social groups, geographical areas and other population categories, that gave fresh impetus to the discussion of the relationship between health and social disparity. A new inquiry led by former Chief Medical Officer Donald Acheson, commissioned within weeks of the election, was tasked with reviewing the latest available information on inequalities on health and developing a cost-effective policy prospectus (Exworthy, 2002). Broad interest in early life influences presented an opportunity for Barker, who was recruited to the scientific advisory committee, while his Southampton colleague Catherine Law assembled input papers and drafted the report. In a letter to Acheson in September 1997, Barker spelled out the policy implications of “inequalities in fetal growth” in terms of improving the “physiological profile” of British children through “long term programming”.^10^ A paper delivered to the committee a few months later similarly invoked animal experiments as providing the decisive evidence of intergenerational effects.^11^ The lasting impact of the Acheson report on policy is contested. Nevertheless, the published report gave significant space to the fetal origins research, which in turn established the prioritization of parents and children as the basis for a national strategy on health inequalities (Acheson, 1998; Bambra et al., 2011).

Although the Acheson report, like Barker’s own project, had been rooted in a longer debate over health inequalities, connections between fetal origins and structural factors of poor health loosened in a “neoliberal” policy climate, with its emphasis on primarily biomedical prevention (Gough, 2013). The “New Labour” government’s emphasis on the “early years” as a “critical period” for tackling intergenerational social exclusion and deprivation was a response to a collapse in consensus on state welfare provision in the 1970s and ‘80s. Part of a broader redefinition of state intervention in terms of prudent and appropriately targeted long-term investment in human capital and increasing equality of opportunity, such wide-ranging measures as the flagship “Sure Start” programme embraced a spectrum of services for the early years and aimed to produce outcomes spanning education, health, social inclusion and community development (Clarke, 2006). By spelling out clear biological and developmental pathways that can be targeted for intervention, research on early life programming chimed with this reorientation in social policy. The continued significance of early years intervention, and its perceived connection to a range of highly visible social problems from childhood poverty to teenage pregnancy under the Labour government, consolidated the profile and relevance of fetal origins research to policy after 2000 (Asthana & Halliday, 2006; Department of Health, 2003; Spencer & Law, 2007). Alliances with and interest from biomedical researchers and the focus on biological mechanisms further overshadowed concern with social determinants of health.

Meanwhile, the new field and its policy implications started to gain traction internationally. A report of the “first international study group” meeting in Sydney in October 1994, bringing together scientists from the UK, Australia, Canada and New Zealand, demonstrates how quickly fetal physiologists pivoted to reframe their studies of fetal growth in terms of environmental influences (Barker, 1995). The physiological communities of former settler colonies, where agricultural interests long supported animal research on the impact of nutritional modifications upon growth, responded especially enthusiastically. Toronto, Adelaide and Auckland, alongside Southampton, quickly developed into centres of development and promotion of the new field, now known as *fetal origins of adult disease*. Fetal origins found eager collaborators amongst established researchers and centres of human nutrition and metabolism in Britain’s former colonies, principally through a post-war research infrastructure established to investigate chronic disease in colonial populations (Moore, 2016). The MRC Unit in The Gambia, study of long-term consequences of famine in Jamaica, or metabolic research in Pune, India all emerged as significant research sites (Forrester et al., 2012; Green, 2018; MRC Lifecourse Epidemiology Unit, 2022). The selection of Mumbai for the First World Congress, in 2001, confirmed the field’s global ambitions. At the 2003 Second World Congress in Brighton the name was changed to DOHaD to indicate a much broader temporal window–from periconception to weaning–and to emphasise the ambition of addressing the determinants of health as well as causes of disease (Hanson, 2015).

Focusing on the well delineated locus of intervention (“intrauterine environment”), short periods of time and set of measurable variables, DOHaD’s proposition fit well into the dominant model of social policy, which had replaced the postwar broad, state-led efforts to fight poverty, with an approach inspired by neoliberal economic thinkers, where the state provided a basic safety-net but encouraged individual effort (Clark, 2020; Sutcliffe-Braithwaite, 2012; Szreter, 2002). It also aligned with the objective of improving living standards of women and girls, and the political slogans around the health of future generations, which resonated in the global health arena as well as in Britain (Pentecost & Ross, 2019). At the same time, by shrinking the environment from the broad social, and political, community to maternal nutrition, it transferred the responsibility for improvement to the mother, in agreement with Conservative (and conservative) support for individualism and “family values” (Sutcliffe-Braithwaite, 2012). This support continued under New Labour, with policies that further affirmed the construction of mothers and their in utero environments as a risk to the fetus, and prioritized an understanding of environments as biological rather than social (Warin et al., 2011 p. 455).

## Conclusion

The “telescoping” of foci from wider social determinants of disease that was characteristic of post-war social medicine, towards the individual bodies of women, we have suggested, can be understood in terms of the alliance between Barker and the fetal and neonatal physiologists. This mutually beneficial exchange was initiated at the Ciba meeting at Villa Marigola, and consolidated, as well as accelerated, across the 1990s as fetal origins of adult disease, then DOHaD, through extensive collaboration across disciplines. As the connections between Barker’s hypothesis and fetal physiology deepened, the maternal uterine environment became the predominant site of concern, effectively marginalizing everything else: from paternal contribution to the broader social environment and structural determinants of health (Moore & Warin, 2022; Richardson, 2021).

By bringing together scientists from different disciplines with common interests in the influence of early experiences on later development in animals and humans, and in promoting the policy implications of this work, Barker was supremely effective at creating a new constituency of support for this model of disease. In doing so, he traded the language and research style of social medicine for a new set of resources, concepts and approaches from fetal physiology and allied experimental disciplines. For fetal physiologists the collaboration with Barker arrived at an opportune moment. After decades of focusing closely on the maturation of fetal functions and elaborating the notion of an “autonomous fetus”, the field was in search of new questions and relevance.

The encounter, as we have described, was extraordinarily productive but not without criticisms. While many of these were of a methodological nature, demanded more evidence or better mechanistic explanations, others, coming from public health and the social sciences, bemoaned the reduction of social environments to biochemical pathways. Some worried that whereas a previous generation of public health researchers examined how social factors influenced infant and child nutrition and health, “today’s epidemiologists investigate, for example, whether the delayed weaning of infants leads to the programming of 7-alpha-hydroxylase activity” (Kuh & Smith, 1993, p.123). Yet in the twenty-first century this substitution of the social environment with a web composed of components tractable through experimental means has come under criticism not only from social scientists, but also new generations of DOHaD researchers. A recent ethnographic study of two major European centres of DOHaD research has revealed fresh worries over the reductionist representation of the fetal environment (Penkler, 2022). More than 30 years after the Villa Marigola meeting, the question of rebuilding the complexity of the fetal environment–of placing the fetus back into the world–is of pressing concern.

## Footnotes

1 The volume describes the image as “the fetus as depicted by the sixteenth century artist Scipione Mercurio in his work the Comare”. The reference appears to refer to Girolamo Mercurio, *La commare o raccoglitrice dell’eccellentissimo signor Scipion Mercurio: Divisa in tre libri*, Verona: Francesco de’ Rossi, 1642.

2 For an entry point into the historical literature on acclimatization, see Bashford and Tracy (2012).

3 See Arni (2018) for the history of French and German traditions in fetal research in the nineteenth and early twentieth centuries.

4 This material would later become a productive resource for research in the DOHaD programme (Batty et al., 2004; Lawlor et al., 2005).

5 See Porter, 2006 for the history of social medicine in international context.

6 See Dron, 2016, for the general appeal of teratology in the postwar decades. By the 1970s, McKeown had grown more pessimistic about the prospects for preventing congenital malformations, other than through abortion (McKeown, 1976, pp. 27, 150).

7 For similar initiatives beyond Britain, see Fairchild et al. (2007), pp. 144–170;

8 However, the East German scientist Günter Dörner used the term “programming” as early as in the 1970s publications on the impact of perinatal conditions on the risk of atherosclerosis, obesity and diabetes risk. He also proposed the concept of “functional teratogenesis” to describe pathological changes in fetal and neonatal physiology–rather than morphology–caused by environmental influences (Dörner, Rodekamp & Plagemann 2008).

9 Neel developed his thrifty phenotype on the back of his broader musings on the ways in which new environmental pressures directed human evolution. On Neel’s early work on the health of the atomic bomb survivors in Japan, see Lindee (1994); on his physical anthropology and human genetics research in Brazil, see Ventura Santos, Lindee and De Souza (2014).

10 Letter from David Barker to Donald Acheson, 4 September 1997, Independent Inquiry into Inequalities in Health (Acheson Committee): Mother/foetus, infant, child and family: input papers, National Archives JA 8/11.

11 David Barker, *Intergenerational Effects on Inequalities in Health*, 8 March 1998, National Archives JA 8/11.
